# The Greater Saphenous Venipuncture Procedure: A Reliable Phlebotomy Method for Neonates

**DOI:** 10.7759/cureus.82837

**Published:** 2025-04-23

**Authors:** Erik J VerHage, Federico Jimenez Ruiz, Thomas R VerHage, Juan C Roig

**Affiliations:** 1 Division of Neonatology, University of Florida, Gainesville, USA; 2 Department of Anesthesiology, University of Florida, Gainesville, USA; 3 Department of Pediatric Medical Imaging, Nemours Children's Hospital, Orlando, USA

**Keywords:** greater saphenous vein, neonatal intensive care unit (nicu), phlebotomy, point-of-care ultrasound (pocus), preterm neonate, venipuncture

## Abstract

Clinicians in the neonatal intensive care unit (NICU) face considerable challenges when caring for the most critically ill and smallest patients, particularly when blood collection is required from those whose umbilical lines have been removed. Advances in NICU care have led to improved survival rates for this vulnerable population, but their smaller size and clinical fragility make venipuncture increasingly difficult. This paper outlines a reliable and accurate approach to venipuncture using specific anatomical landmarks. While our team at the University of Florida utilized point-of-care ultrasound (POCUS) to confirm procedural accuracy, the primary aim of this publication is to equip clinicians with a practical, landmark-based method for obtaining blood samples. This approach is particularly valuable in cases where phlebotomy is challenging due to patient size, in the absence of central vascular access, or in developing countries and units without access to POCUS machines.

## Introduction

Infants in the neonatal intensive care unit (NICU) require frequent lab draws [1]. For patients with vascular access that can draw blood (i.e., umbilical catheters, tunnel central catheters, and peripheral arterial lines), obtaining laboratory samples is readily available; however, as units strive to decrease central line-associated bloodstream infections (CLABSIs), indwelling catheters are being pulled earlier, and the frequency of central line access for laboratory sampling is being minimized whenever possible [2], making providers more reliant on venipuncture, arterial puncture, or heel prick for laboratory samples. Peripheral venous or arterial punctures can present challenges, particularly as NICUs manage increasingly premature infants, encounter repeated failed attempts, or face the demands of frequent routine laboratory testing. Furthermore, neonatologists are often faced with situations such as a sick infant with poor peripheral perfusion in need of immediate laboratory assessment or an extremely premature infant whose umbilical lines have been removed but needs urgent lab draws. The growing need for specialized genetic testing [3] further compounds these challenges, as such testing often necessitates large-volume blood draws that may be difficult to obtain through a single venipuncture.

In 1978, Plaxico and Bucciarelli introduced a landmark-based approach to greater saphenous (GS) venipuncture, highlighting several advantages of this site. These included the vessel’s size, which facilitates the rapid collection of relatively large blood volumes; the elimination of the need to palpate and potentially contaminate the skin to locate the vessel; the avoidance of complications associated with femoral artery and external jugular vein puncture; and the use of consistently reproducible landmarks, which make the procedure straightforward and reliable [4]. However, in our experience, some clinicians remain hesitant to perform this technique due to its proximity to the femoral triangle and the associated risks of needle malposition. The original publication did not include reference images or clear anatomic landmarks to guide clinicians in performing this procedure with reproducible success. Since 1978, there has been limited subsequent literature addressing this method of venous sampling.

The aim of this publication is to equip neonatal providers without access to point-of-care ultrasound (POCUS) with a reliable, landmark-based approach to venipuncture, particularly in cases where other methods of vascular access have failed or are unavailable. While POCUS was utilized in this study to confirm needle tip position and demonstrate procedural safety, the technique itself is routinely performed without the need for real-time imaging guidance. It is important to emphasize that this approach is not intended for routine laboratory sampling or frequent use of the GS vein.

## Technical report

Description of the anatomy

**Figure 1:**
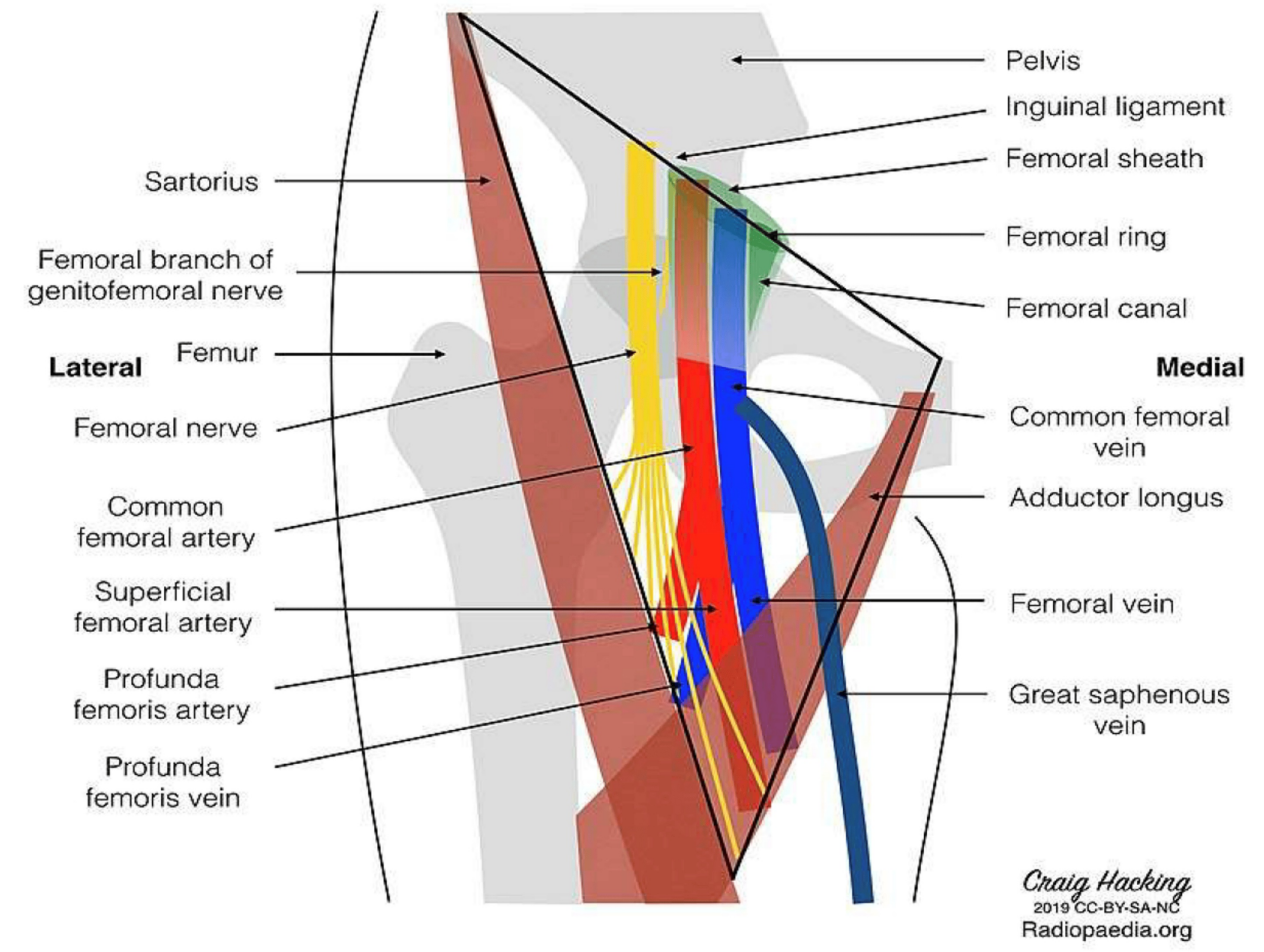
Illustration of the structures contained within the femoral triangle Of interest, the GS vein is the last branch of the femoral vein at the level of the femoral triangle, coursing superficially to the adductor longus. GS: greater saphenous Image obtained from Hacking (2019) [5]

**Figure 2:**
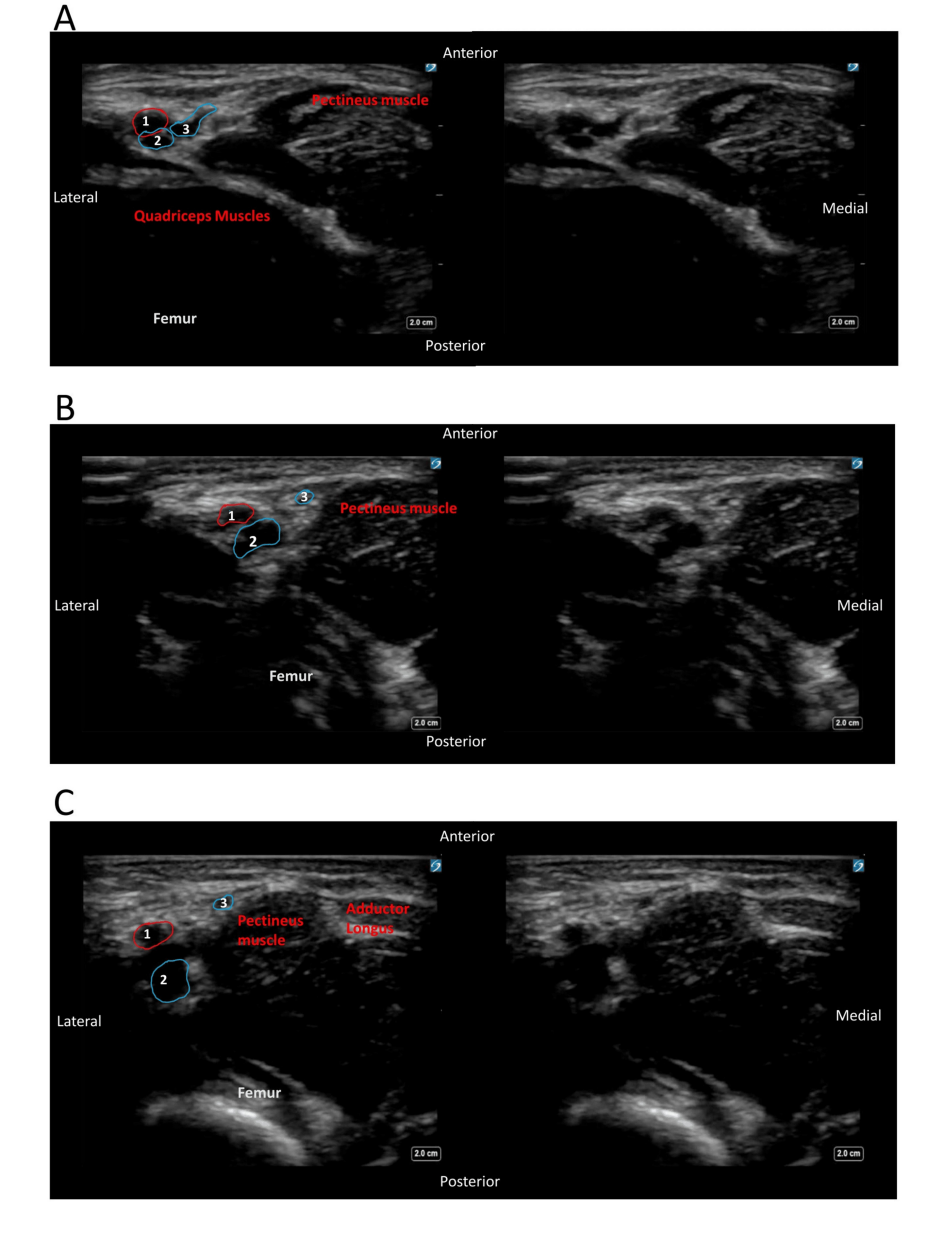
Ultrasound images of the vessels in the right femoral triangle in a proximal-to-distal sweep in the axial plane, with an annotated image on the left and original image on the right (A) Most proximal view demonstrating branching of the GS vein from the common femoral. (B) Middle view of the sweep with the GS separating from the femoral vein superficial to the pectineus muscle. (C) Distal view of the GS separation from the femoral vein, traversing superficial to the pectineus muscle with the adductor longus — medial border of the femoral triangle — coming into view. 1: femoral artery; 2: femoral vein; 3: GS vein GS: greater saphenous

The anatomical boundaries of the femoral triangle are as follows: superiorly, the base of the triangle is formed by the inguinal ligament; medially, it is defined by the lateral edge of the adductor longus muscle; and laterally, it is bordered by the medial aspect of the sartorius muscle (Figure 1). The femoral triangle contains several important structures, including the femoral nerve, common femoral artery, common femoral vein, and GS vein. These structures are arranged laterally to medially in the following order: nerve, artery, and vein, which can be remembered using the acronym “NAVL.” In the frog leg position, the GS vein becomes relatively accessible for phlebotomy. This vein is notable for its drainage into the common femoral vein just before it enters the abdomen (Figure 2).

Description of the procedure

The study protocol was approved by the University of Florida Institutional Review Board (approval number 202400041). Informed consent was obtained from the families of the patients included in this publication, covering the performance of the phlebotomy, the use of photographs, and the use of ultrasound imaging. The procedure was performed by neonatologists, while ultrasound imaging was conducted by a pediatric anesthesiologist with expertise in POCUS. All images were subsequently reviewed by a pediatric radiologist to ensure accuracy and consistency. Venipuncture was performed using a 23-gauge butterfly needle or straight needle with a 3-mL syringe.

**Figure 3:**
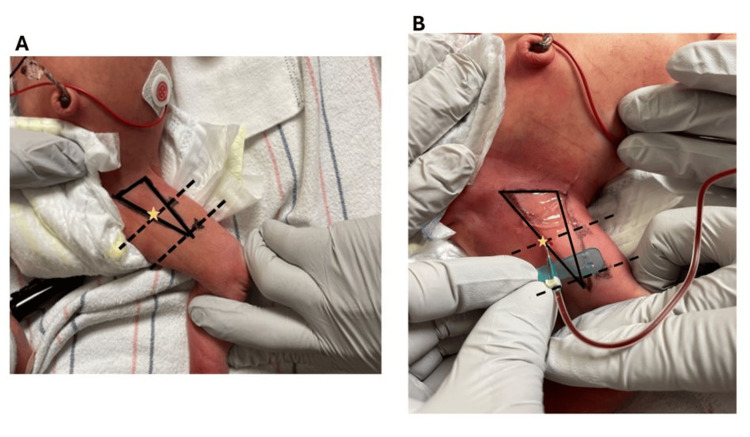
Annotated landmarks during the procedure (A) The patient’s thigh placed in a “frog leg” position with the landmarks of the femoral triangle marked. Thigh divided into thirds. (B) Insertion site of the needle on the lateral edge of the adductor longus muscle angled at 45 degrees and aimed toward the patient’s umbilicus. Solid lines: borders of the femoral triangle; dashed lines: thigh divided into thirds; star: site of needle introduction where the adductor longus meets the proximal third line

This procedure does not require the use of a tourniquet or sedation. It is typically approached in a manner consistent with standard phlebotomy practices, with the recommended use of oral sucrose or expressed human milk to reduce periprocedural pain and stress. The patient is positioned supine with the hips flexed and abducted in a “frog leg” position. Typically, an assistant stabilizes the patient’s torso, while the proceduralist secures the knee with one hand. The femoral triangle is identified and aseptically prepared (Figure 3A). The patient’s thigh is divided into three equal segments, as indicated by dashed lines. After this, the proceduralist uses the hand not securing the knee to manipulate and advance the needle into the puncture site, which is located within the femoral triangle at the junction between the proximal third and the middle third of the thigh, as illustrated in Figure 3B.

**Figure 4:**
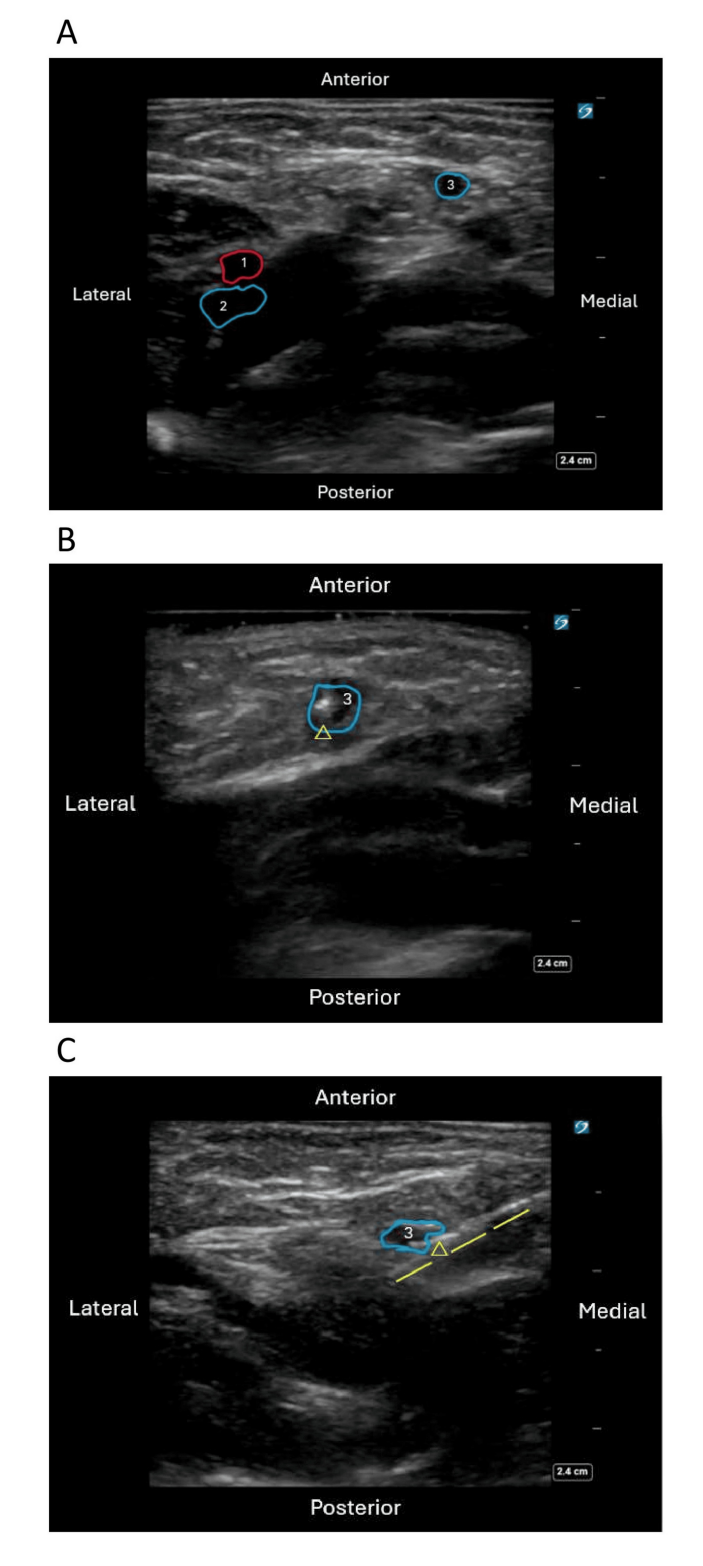
Ultrasound confirmation of needle tip location (A) Redemonstration of the anatomy of the GS vein in relation to the femoral artery and vein in the axial plane. (B) Needle tip in the GS vein perpendicular to the ultrasound probe in the axial plane. (C) Sagittal plane view of the needle tip in the GS vein. 1: femoral artery; 2: femoral vein; 3: GS vein GS: greater saphenous

The phlebotomy needle should be inserted laterally at the edge of the adductor longus muscle at a 45-degree angle, directed toward the patient’s umbilicus (Figure 3). Once the needle bevel is subcutaneous, slight vacuum pressure should be applied while gradually advancing the needle along the specified trajectory until blood return is observed. At this point, the needle should not be advanced further, as the vessel accessed should be the GS vein. It is crucial to maintain the needle angle at 45 degrees, consistently oriented toward the patient’s umbilicus. The vascular anatomy was confirmed using POCUS post-procedurally during the procedure to verify that the GS vein was consistently accessed using these landmarks (Figure 4).

## Discussion

In this study, we described a landmark-based approach for reliable venous sampling in neonates. POCUS was only used to confirm needle tip location and provide reassurance to clinicians given the proximity to the femoral triangle and its associated vessels and nerves. As with any procedure, potential risks include bleeding, infection, and thrombosis. Prior to performing the procedure, clinicians should carefully evaluate the risks and benefits and engage in shared decision-making with the family. While our findings demonstrate that adherence to the landmarks described in this study minimizes the risk of needle malpositioning and associated injury to the femoral nerve, artery, or vein, anatomical variations may still pose a risk. In neonates with significant coagulopathy, there is an elevated risk of complications such as extensive hematoma formation due to bleeding into surrounding tissues. A notable complication of this procedure, an acquired arteriovenous malformation, was reported in 1985 [6]; however, this occurred during an era when GS vein venipuncture was routinely used for most laboratory samples in that unit. We do not advocate for the routine use of this technique for daily laboratory sampling but suggest its consideration in select cases where peripheral venous access proves insufficient. The objective of this report is to assess the feasibility of GS vein cannulation using an anatomical landmark-based technique without the aid of real-time ultrasound guidance. Ultrasound was utilized post-procedurally to confirm needle placement. By demonstrating the reliability of this technique, we aim to equip clinicians, particularly those practicing in resource-limited settings where POCUS devices may be scarce, with a practical method for obtaining vascular access in a larger-caliber vein to facilitate safe and effective blood sampling. Further studies to report success rate in a large cohort of patients may be warranted; however, anecdotally, this has been frequently used in our unit for many years with a high level of success.

In addition to demonstrating the relative safety and efficacy of the procedure, particularly in limited resource settings, this method of venipuncture may assist in enabling reliable access to a larger-caliber vessel, potentially reducing the number of needle sticks required. Elevated scores on the Neonatal Infant Stressor Scale have been associated with adverse neurodevelopmental outcomes [7]. In recognition of this, the EPIPPAIN study emphasized the importance of adopting strategies to minimize needle stick attempts in neonatal care [1]. While this venipuncture method may not substantially reduce the overall burden of painful procedures in the NICU to achieve downstream outcomes such as improved brain development, it remains imperative for clinicians caring for this vulnerable patient population to carefully consider the impact of each painful procedure and strive to minimize exposure to adverse stimuli.

## Conclusions

This study presents a landmark-based approach for GS vein access in neonates, offering a practical and reliable method for venous sampling when peripheral access is limited. While not recommended for routine use, this technique may reduce needle sticks and support safer access in select cases, particularly in resource-limited settings. Further studies are warranted to validate safety and efficacy in larger cohorts.
